# Introducing the neurosciences section of the journal of translational medicine

**DOI:** 10.1186/1479-5876-9-117

**Published:** 2011-07-21

**Authors:** Luis F Alguacil

**Affiliations:** 1 Translational Research Unit, Hospital General de Ciudad Real, Ciudad Real, Spain and Laboratory of Pharmacology and Toxicology, San Pablo CEU University, Madrid, Spain

## 

'We must seriously think about proceeding further than looking for cures just for rodents". These ironic words were recently pronounced in a public forum by a medical doctor devoted to basic research, who decided to move from experimentation in rat arteries to human vessels in her search for new targets for cardiovascular diseases. Scientists dealing with basic neuropsychiatric research must seriously examine this question and another one even more critical: are we looking for cures for diseases in biological systems not willing to suffer them in nature? In other words: can we consider spontaneous dementia, anorexia nervosa, addiction or schizophrenia specific human diseases? If so... is it intelligent trying to progress in the knowledge of these pathologies on the basis of home-made, artificial animal models? Must we persist on this way or center exclusively on human experimentation?

The bench to bedside two-way travel is always problematic, but it appears especially difficult when dealing with neuropsychiatric disorders where psychosocial variables seem to play an essential role, both in the emergence and maintenance of the problem. This fact extremely complicates laboratory modeling. It is clear that the fast development of the neurosciences along the XX century has led to important medical progress, enabling therapists to use modern, advanced tools (i.e., rationale-based designed drugs instead of serendipity-based old remedies). However, a significant gap still persists between basic science and medical applications for the patients, in part due to the shortage (sometimes absence) of preclinical models with enough face, construct and predictive validity. This may explain why we keep unable to find ways to defeat old, classical barriers, i.e. the widely known 3/4-week lapse for the onset of antidepressant actions or the persisting 30% of patients still refractory to the available antidepressants. Finding new ways to overcome this bottleneck seems therefore a major challenge for neuroscientists.

When significant advances are finally achieved in basic science, and even in the case that they look promising in randomized clinical trials, prominent barriers still remain to prevent fast translations into clinical practice. These are the targets of the so-called T2 translational research, as defined by the American Institute of Medicine's Clinical Research Roundtable. T2 research deals with problems such as cost-effectiveness and feasibility of implementation in health care systems. The need for promoting T2 research and its coupling with new developments from basic neuroscience has been recently qualified as "urgent" by NIMH investigators working on psychotic disorders, especially worried about the outcome of extremely vulnerable populations. Besides psychiatrists, neurologists have also expressed the opportunity of encouraging all the steps of translational research from basic science to the clinics and backwards, a process which is expected to render promising results in prioritized research areas (stroke, headache, multiple sclerosis, epilepsy and dementias, in the case of the US).

Translational research on neuropsychiatric disorders is less developed than in other areas (i.e. oncology), but it is an emerging issue. Any new tool for the dissemination of results in the field can greatly help to progress in the achievement of new solutions for the neuropsychiatric patient, and this is the main goal of the new Neurosciences Section of the Journal of Translational Medicine. We hope to encourage publications and promote the increase of knowledge on the subject by collecting original articles together in this new specific section.

Luis F. Alguacil, Section Editor Neurosciences

